# Long-acting prescriptions and therapy for HIV-1 from market launch to the present in Germany (May 2021 to December 2023)

**DOI:** 10.3389/fpubh.2024.1404255

**Published:** 2024-05-30

**Authors:** Daniel Schmidt, Christian Kollan, Matthias Stoll

**Affiliations:** ^1^Department of Infectious Disease Epidemiology, Robert Koch Institute, Berlin, Germany; ^2^Hannover Medical School, Hannover, Germany

**Keywords:** cabotegravir (CAB), rilpivirine (RPV), long-acting (LA) HIV therapy, HIV treatment, injectables, Germany, CARLA

## Abstract

**Background:**

In Europe, the combination of cabotegravir (CAB) with rilpivirine (RPV) has been approved as a dual injection long-acting (LA) therapy for the treatment of human immunodeficiency virus type 1 (HIV-1) infections in adults since December 2020. Studies have shown that between 36 and 61% of people living with HIV (PLWHIV) prefer LA therapy. However, there are no real-world data on the number of people receiving LA therapy, in Germany or internationally. The aim of this study was to assess the current situation and trends in usage of LA therapy for the treatment of HIV-1 in Germany.

**Methods:**

Based on pharmacy prescription data derived from Insight Health, the monthly number of prescriptions for oral CAB, CAB-LA, and RPV-LA over the entire period of availability in Germany was analyzed and evaluated (May 2021 to December 2023). The number of 1st and 2nd initiation injections and subsequent maintenance injections was calculated on the basis of the prescriptions for oral CAB initiation.

**Results:**

The bimonthly schedule resulted in two growing cohorts from September 2021 with an estimated 14,523 CAB-LA prescriptions over the entire period. Accordingly, in December 2023, there were approximately 1,364 PLWHIV receiving LA therapy, of whom 1,318 were receiving maintenance therapy. Only treatments with bimonthly regimens were carried out. Accounting for people not covered by statutory health insurance (~13%), a total of ~1,600 PLWHIV were receiving LA therapy in Germany in December 2023. The average rounded annual cost of therapy in 2023 was €11,940 (maintenance therapy with initiation) and €10,950 (maintenance therapy without initiation).

**Conclusion:**

To our knowledge, this is the first study of real-world use and number of people receiving LA therapy. A strength of our study is the nearly complete coverage of people with statutory health insurance in Germany. The predicted demand for LA therapy does not match the actual number of people receiving LA therapy. Although the number of PLWHIV receiving LA therapy increased steadily, they accounted for just under 2% of the estimated total number of people receiving HIV therapy in Germany in 2023, almost 2 years after the market launch. No significant increase in prescriptions is expected; on the contrary, the trend is leveling off and is unlikely to change drastically in the near future. Hence, the need for this mode of therapy in Germany appears to be limited. Follow-up studies at regular intervals on the further course would be useful and are recommended, as well as investigations into the possible reasons for the slow uptake to inform public health experts and possibly broaden treatment options.

## 1 Background

In Europe, cabotegravir (CAB) in combination with rilpivirine (RPV) has been approved for dual injection therapy for the treatment of human immunodeficiency virus type 1 (HIV-1) infections in adults since December 2020. The prerequisite is that individuals are virologically suppressed (HIV-1 RNA < 50 copies/ml) under a stable antiretroviral therapy (ART) regimen and show no current or previous signs of viral resistance and no previous virological failure against agents of the nonnucleoside reverse transcriptase inhibitor and integrase strand transfer inhibitor class (1). The market launch in Germany occurred in May 2021 (2, 3).

Two application scenarios are possible. The one-month dosing regimen includes 400 mg CAB and 600 mg RPV, and the two-month dosing regimen includes 600 mg CAB and 900 mg RPV. In both dosing regimens, the first initiation injection is given with a combination of 600 mg CAB/900 mg RPV, followed by a second initiation injection 1 month later. The second initiation injection and the maintenance injections can each take place up to 7 days before or after the planned target date (1). This mode of treatment is also referred to as “long-acting” (LA) therapy.

Prior to the initiation of injections, LA therapy with this drug combination should be initiated in the first month with a once-daily oral tablet dose of 30 mg CAB and 25 mg RPV. However, this oral initiation phase is not mandatory (1).

The use of long-acting HIV drugs offers advantages in the field of HIV treatment and prevention. However, there are also challenges in terms of manageability, drug safety, possible development of resistance, storability, distribution channels and cost considerations.

Studies on the willingness to switch to LA therapy indicate that 36 to 61% of people living with HIV (PLWHIV) prefer LA therapy (4–6). However, there are no real-world data on the number of people receiving LA therapy, in Germany or internationally. The aim of this study was to assess the current situation and trends in usage of LA therapy with CAB-LA/RPV-LA for the treatment of HIV-1 in Germany after more than 2 years of market availability.

## 2 Methods

Based on pharmacy prescription data from Insight Health, the monthly number of prescriptions for oral CAB, CAB-LA, and RPV-LA over the entire period of availability in Germany was analyzed and evaluated (May 2021 to December 2023). Based on the oral CAB prescriptions and dosing regimens, the number of initiations and the number of PLWHIV receiving LA therapy were estimated and compared with the actual CAB-LA prescriptions. The number of CAB-oral prescriptions was added to the next month for the 1st and 2nd initiations; for maintenance therapy, it was successively added in two monthly steps. In this scenario, theoretically, no patients discontinued LA therapy.

The prescription data provider Insight Health reports a coverage of >99% of prescriptions in the statutory health insurance sector. In Germany, health insurance is compulsory and about 87% of citizens are covered by statutory health insurance (7, 8). Therefore, the data represent ~73.6 million insured persons in Germany (9).

After accounting for the proportion of people who are not covered by statutory health insurance (~13%), the total number of PLWHIV receiving LA therapy was estimated for Germany.

Furthermore, for the CAB-LA prescriptions, the regional distribution based on the federal state location of the prescribing practitioner was compared with the estimated regional distribution of the proportion of people receiving HIV therapy in Germany. The development of the average costs according to the pharmacy retail price over the entire period was evaluated and compared with a comparable oral HIV dual therapy with dolutegravir and RPV.

## 3 Results

### 3.1 Estimate of the number of patients treated

Over the entire period, 1,427 monthly packages of CAB-oral (30 tablets of 30 mg each) were recorded. In addition, 14,616 prescriptions for CAB-LA 600 mg and 14,765 prescriptions for RPV-LA 900 mg as an injection suspension were counted (a difference of 149 prescriptions). The corresponding values are shown in Table 1.

**Table 1:** Number of prescriptions of CAB-LA, RPV-LA, or CAB-oral and estimated 1st and 2nd initiation, maintenance therapies and PLWHIV under long-acting (LA) therapy within the statutory health insurance in Germany.

		Insight health data			Estimation based on CAB-oral prescriptions					
Year	Month	RPV-LA prescriptions	CAB-LA prescriptions	CAB-oral prescriptions	1. Initiation injection	2. Initiation injection	Maintenance cohort 1	Maintenance cohort 2	Estimated CAB-LA prescriptions	PLWHIV under LA
2021	5	21	23	63					0	
	6	78	79	97	63				63	97
	7	184	184	73	97	63			160	170
	8	199	197	98	73	97			170	268
	9	241	240	76	98	73	63		234	344
	10	296	298	64	76	98		97	271	408
	11	300	294	61	64	76	136		276	469
	12	353	352	49	61	64		195	320	518
2022	1	367	368	55	49	61	212		322	573
	2	382	378	50	55	49		259	363	623
	3	446	445	46	50	55	273		378	669
	4	422	422	41	46	50		308	404	710
	5	463	456	46	41	46	328		415	756
	6	485	480	36	46	41		358	445	792
	7	471	467	41	36	46	374		456	833
	8	530	524	35	41	36		399	476	868
	9	529	528	33	35	41	420		496	901
	10	474	471	34	33	35		435	503	935
	11	503	495	31	34	33	461		528	966
	12	450	449	25	31	34		470	535	991
2023	1	538	535	35	25	31	494		550	1,026
	2	526	519	45	35	25		504	564	1,071
	3	559	555	39	45	35	525		605	1,110
	4	595	585	26	39	45		529	613	1,136
	5	636	628	24	26	39	560		625	1,160
	6	552	542	33	24	26		574	624	1,193
	7	630	624	27	33	24	599		656	1,220
	8	695	685	35	27	33		600	660	1,255
	9	661	648	28	35	27	623		685	1,283
	10	731	718	25	28	35		633	696	1,308
	11	713	695	34	25	28	650		703	1,342
	12	735	732	22	34	25		668	727	1,364
Total		14,765	14,616	1,427	1,405	1,371			14,523	
Difference in prescriptions between estimation based on CAB-oral vs. Insight Health CAB-LA prescriptions.									−93	

CAB-LA 400 mg and RPV-LA 600 mg were not prescribed during the study period. Accordingly, only treatments with the bimonthly regimen were carried out. The monthly dose is no longer available in Germany.

**Figure 1:**
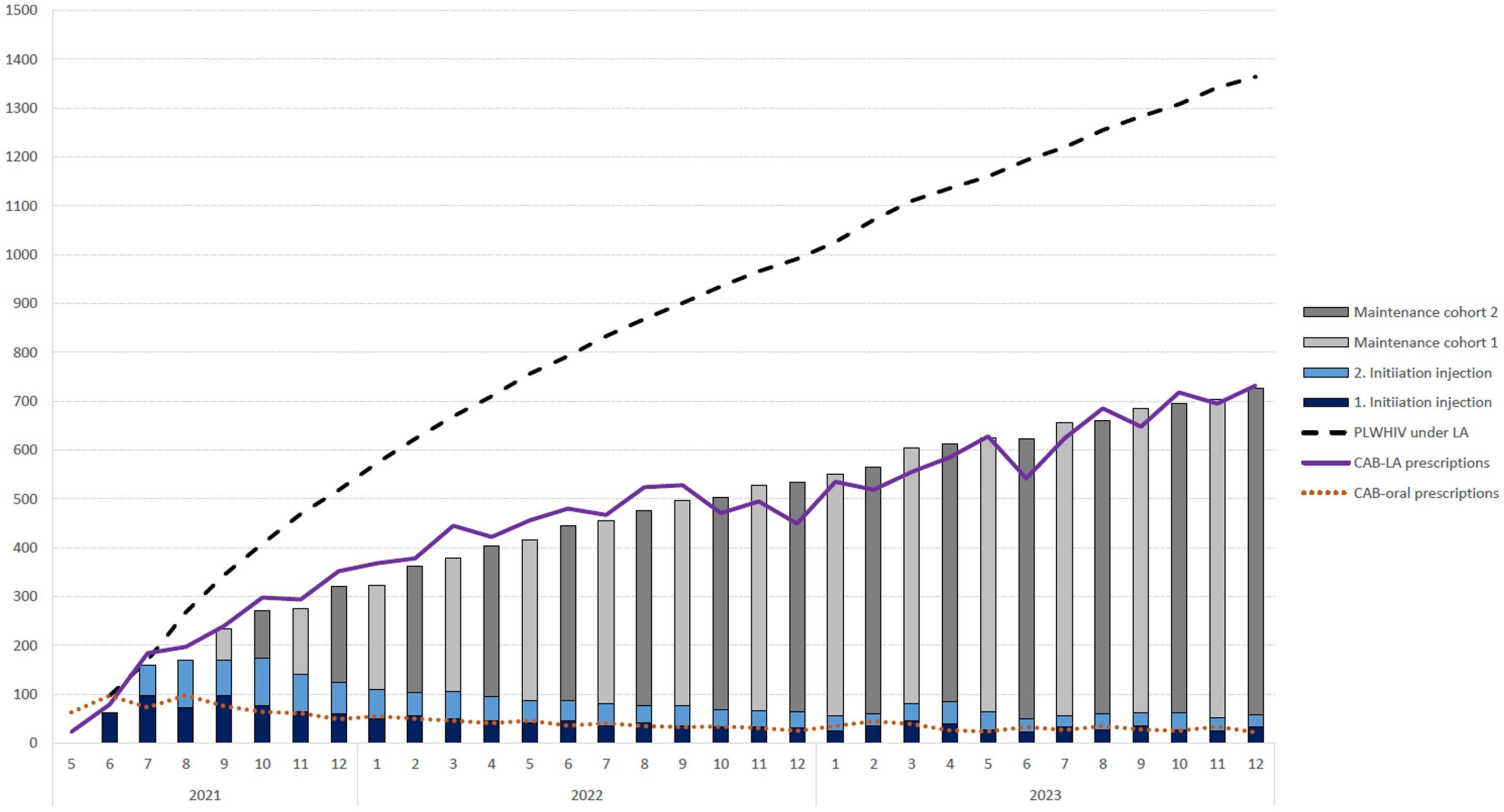
Number of monthly prescriptions for oral cabotegravir initiation (CAB-oral prescriptions) and the resulting 1st and 2nd initiation injection, maintenance therapy cohorts and resulting number of people living with HIV (PLWHIV) receiving long-acting (LA) therapy within the statutory health insurance in Germany over time.

The number of 1st and 2nd initiation injections and subsequent maintenance injections was calculated on the basis of the prescriptions for oral CAB initiation. The bimonthly schedule resulted in two growing cohorts beginning in September 2021, each of which received their maintenance injection in a month with an even or odd number. This resulted in 14,523 estimated prescriptions over the entire period, which means that in December 2023, there were approximately 1,364 PLWHIV receiving LA therapy, of which 1,318 were receiving maintenance therapy. This trend is shown in Figure 1.

Accounting for a proportion of 13% of people who are not covered by statutory health insurance, a total of ~1,600 PLWHIV were receiving LA therapy in Germany in December 2023.

The average rounded annual therapy costs were € 14,210 (maintenance therapy with initiation) and € 13,150 (maintenance therapy without initiation) in 2021, € 13,100 and € 12,050 in 2022 and € 11,940 and € 10,950 in 2023.

The costs for comparable oral HIV dual therapy with dolutegravir and RPV were constant at € 10,270 over the entire comparison period.

### 3.2 Distribution in the federal states

Table 2 shows the proportionate distribution of CAB-LA prescriptions in the federal states as well as a comparison of the estimated distribution of HIV patients receiving therapy in Germany with the distribution of CAB-LA prescriptions in the federal states. Comparatively high proportions of CAB-LA prescriptions (> 5%) were found in North Rhine-Westphalia, Berlin, Bavaria, Hamburg and Baden-Württemberg.

**Table 2:** Number of CAB-LA prescriptions, proportionate distribution of CAB-LA prescriptions in the federal states, estimated distribution of people receiving HIV therapy in Germany and proportions and comparison with the distribution of CAB-LA prescriptions in the federal states.

Federal state (FS)	CAB-LA prescriptions	Proportion in federal state	People under HIV therapy in Germany in 2023	Proportion in federal state	Difference between proportion of CAB-LA prescriptions and people under HIV therapy
North Rhine-Westphalia	4046	27.7%	18393	21.9%	6%
Berlin	4009	27.4%	12120	14.4%	13%
Bavaria	2151	14.7%	11589	13.8%	1%
Hamburg	1392	9.5%	6900	8.2%	1%
Baden-Württemberg	1011	6.9%	13,077	15.5%	−9%
Lower Saxony	538	3.7%	3,200	3.8%	0%
Hesse	510	3.5%	6,050	7.2%	−4%
Saxony	288	2.0%	2,839	3.4%	−1%
Saxony-Anhalt	238	1.6%	1,255	1.5%	0%
Bremen	99	0.7%	1765	2.1%	−1%
Rhineland-Palatinate	81	0.6%	2,158	2.6%	−2%
Thüringen	73	0.5%	627	0.7%	0%
Mecklenburg Western Pomerania	63	0.4%	1,340	1.6%	−1%
Schleswig-Holstein	59	0.4%	1861	2.2%	−2%
Saarland	56	0.4%	861	1.0%	−1%
Brandenburg	1	0.0%	85	0.1%	0%
Total	14,615	100%	84120	100%	

**Figure 2:**
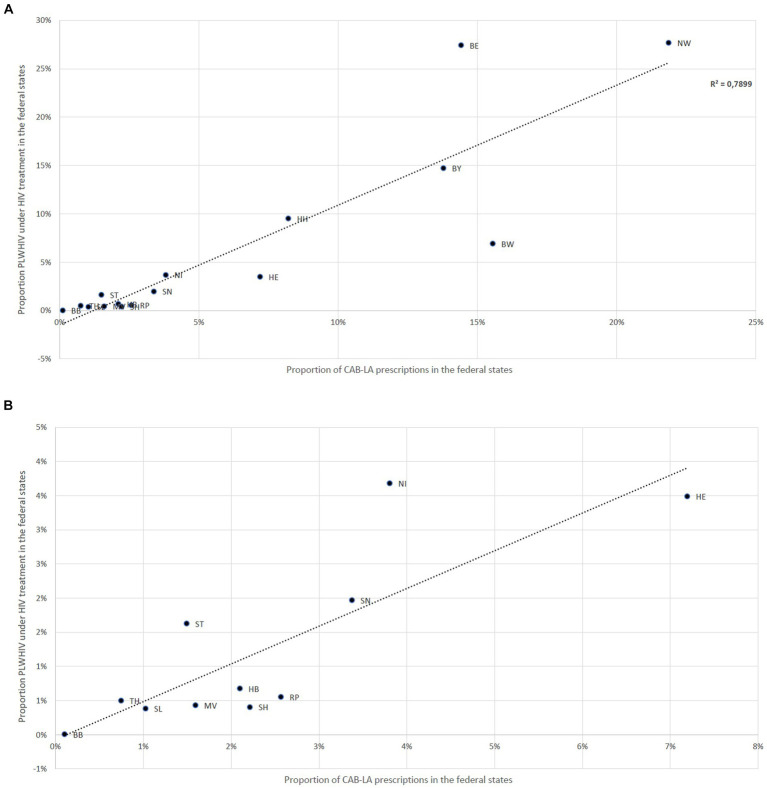
**(A)** Deviations between the proportion of CAB-LA prescriptions in the federal states and the estimated proportion of people receiving HIV therapy in the federal states; Baden-Württemberg (BW), Bavaria (BY), Berlin (BE), Brandenburg (BB), Bremen (HB), Hamburg (HH), Hesse (HE), Mecklenburg-Western Pomerania (MV), Lower Saxony (NI), North Rhine-Westphalia (NW), Rhineland-Palatinate (RP), Saarland (SL), Saxony (SN), Saxony-Anhalt (ST), Schleswig-Holstein (SH), Thuringia (TH). **(B)** Deviations between the proportion of CAB-LA prescriptions in the federal states and the estimated proportion of people receiving HIV therapy in the federal states; only federal states with a proportion of CAB-LA prescriptions of less than 4% on all CAB-LA prescriptions shown; Brandenburg (BB), Bremen (HB), Hesse (HE), Mecklenburg-Western Pomerania (MV), Lower Saxony (NI), Rhineland-Palatinate (RP), Saarland (SL), Saxony (SN), Saxony-Anhalt (ST), Schleswig-Holstein (SH), Thuringia (TH).

Figure 2 illustrates the proportions of CAB-LA prescriptions in the federal states compared to the estimated distribution of the proportion of people receiving HIV therapy in the federal states. A deviating distribution (> ± 3%) was found in Berlin and North Rhine-Westphalia, where the CAB-LA prescription share was higher, and in Baden-Württemberg and Hesse, where the CAB-LA prescription share was lower.

## 4 Discussion

The number of PLWHIV receiving LA therapy increased steadily over the entire period. In December 2023, there were ~ 1,400 PLWHIV with statutory health insurance receiving LA therapy in Germany. After accounting for the proportion of people not covered by statutory health insurance, the total number of PLWHIV receiving LA therapy in Germany was ~1,600 in December 2023. Since the innovative form of treatment with LA is presumably not available to uninsured persons and prisoners in the same way as to privately insured persons, the share of 13% not covered by statutory health insurance may overestimate the actual total number of PLWHIV receiving LA treatment in Germany. Although the number of PLWHIV receiving LA therapy increased steadily, they accounted for just under 2% of the estimated total number of people receiving HIV therapy in Germany in 2023, almost 2 years after the market launch. In contrast, studies suggested a much higher demand and uptake, of around one-third to two-thirds of PLWHIV (4–6). The number of LA treatment initiations increased to ~100 per month in the first 4 months and decreased to approximately half by mid-2022. Since then, a slight downward trend has been observed, so that by the end of the observation period, the number of LA treatment initiations was approximately 30 per month. This trend is unlikely to change drastically in the near future. With an estimated 2,500 persons newly initiating HIV therapy per year, the need compared to the forecasted demand for this mode of therapy in Germany appears to be limited in 2023 (4–6, 10). The development and implementation of LA concepts in infectiology is still a novel area of clinical research and practice. The concept of LA treatment is basically simple and plausible, as it simplifies HIV therapy and is low threshold. However, there are numerous challenges, including questions of manageability, drug safety, possible development of resistance, storability, distribution channels and cost considerations, which have already been discussed in more detail elsewhere (11, 12). The questions still unanswered about this new option may be a reason for PLWHIV and medical practitioners to stick with the long-established options of oral ART in case of doubt. The reasons for the relatively slow increase in everyday practice in Germany are therefore presumably manifold and predominantly not medical reasons in the narrow sense.

### 4.1 Application

On the one hand, parenteral administration, which is now only necessary every 1 or 2 months, is advantageous for PLWHIV who either cannot tolerate oral ART, cannot reconcile it with their professional or private lives or who are concerned about unintentional disclosure of their HIV infection. On the other hand, LA therapy involves high requirements and restrictions in terms of flexibility due to its application (12).

- Application interval: with the two-month interval of the injections, the treatment intervals are certainly still at the lower end of the preference for PLWHIV. This assumption is clearly supported by the fact that the LA option for 1 month of administration, which was theoretically also available in Germany, has not yet been prescribed. After all, the limited possibility of postponing the due injection by (only) plus/minus 1 week for PLWHIV requires careful and long-term forward-looking planning.
- Form of administration: the approval of CAB-LA and RPV-LA is subject to (i) administration by a health care professional and (ii) safe intramuscular (gluteal) injection (1).

It is therefore not (yet) possible for PLWHIV to administer the treatment themselves, and they are therefore tied to specialized medical institutions that offer LA therapy (12). In Germany, these are almost exclusively HIV specialty care centers and outpatient clinics (13). Paradoxically, this usually means that these HIV focal points must be visited more frequently when patients are receiving LA therapy (every 2 months) than when patients are receiving conventional, oral ART (every 3 months or even less frequently) (12). In addition to more frequent doctor visits, bimonthly LA therapy results in three times greater copayments at pharmacies than do three-monthly packages of an oral single-tablet ART regimen, and one-monthly LA administration even results in six times greater copayments.

Administration by intramuscular LA injection into the outer thigh, which has now been investigated as an alternative, could be an option in the future to enable trained PLWHIV to self-apply (14).

### 4.2 Cooling

The approval of LA therapy was subject to the condition of a strictly monitored supply of the substance within a well-documented cold chain from production to injection, at least for RPV-LA (1, 15). Maintaining a cold chain with many stations may sound trivial, but in practice, it is prone to failure. The refrigerated medicine is sent to the pharmacy via the wholesaler or directly via the pharmaceutical manufacturer. From there, a medicine usually goes to the PLWHIV, where there is probably the highest risk of the cold chain being interrupted. The PLWHIV take it to the (medical) treatment center if the medicine is not delivered directly from the pharmacy to the practice. If this process is disrupted, the medicine may have to be completely discarded or destroyed (15). Disruptions in the process, which cannot always be ruled out, can therefore have extremely drastic consequences for PLWHIV. Refrigeration and application by expert staff also make spontaneous visits to the HIV specialty care center or pharmacy more difficult – for example, to shorten the originally planned LA interval (permissibly) by a week due to a short-term commitment.

### 4.3 Stop regulations

ART should neither be interrupted nor discontinued without replacement; otherwise, there is a high health risk due to virological rebound or the development of resistance (16). For LA therapy in particular, the so-called pharmacological tail results in months of subinhibitory plasma concentrations after discontinuation and thus a greatly increased risk of HIV resistance (17). This can be effectively countered with bridging strategies, i.e., continuation with oral ART (18). However, this option is unlikely to be implemented in the case of so-called unstructured treatment interruption, regardless of whether this interruption was due to reasons related to PLWHIV or unexpected external circumstances. This concern about a lack of strategy in the event of an unplanned therapy interruption can also be a reason why both PLWHIV and health care professionals prefer oral ART over the LA option.

### 4.4 Dispensing rights

The areas of activity of pharmacies and human medicine are strictly separated in most countries. In Germany, only pharmacists, not doctors, have the right to dispense medicines prescribed by a doctor (19). Patients’ freedom to choose the pharmacy or doctor’s practice they trust must not be restricted at any time. In this respect, structured supply chains between specialized pharmacies and HIV specialty care centers could be legally contestable. On the other hand, it is usually only these specialized centers that are willing and have the infrastructure to guarantee the supply of LA. Since the services offered are only economically viable above a certain sales threshold and LA drugs are rarely in demand in Germany due to the low HIV prevalence, there is a higher barrier to access for the subgroup of PLWHIV in Germany who live in regions with a particularly low HIV prevalence.

These reasons could also explain the differences found between the proportion of people receiving HIV therapy and the proportion of PLWHIV receiving LA therapy in the federal states. In federal states with large urban centers with a known high HIV prevalence and a correspondingly higher density of HIV specialty care centers, LA therapies are more strongly represented, whereas they are underrepresented in equally populous but less structured (e.g., rural) areas. This is a strong indication of a greater potential for prescribing LA in Germany, provided that access to this therapy would be more low threshold than before. A significant proportion of the existing hurdles could be addressed more effectively and presumably more sustainably at an administrative and regulatory level than through additional investment in specific medical care infrastructures.

However, meanwhile, the German drug price alone does not appear to be a limiting factor. Although at the beginning of LA therapy in 2021 and 2022, the pharmacy retail price of LA maintenance therapy was approximately 20 to 30% greater than that of a comparable dual therapy with Dolutegravir combined with RPV. At the end of the study period in 2023, the price of LA maintenance therapy differed only insignificantly from that of an oral comparator therapy. The price of LA therapy also decreased by 17% over the observation period.

The use of CAB-LA for pre-exposure prophylaxis (PrEP) of HIV infection is a promising option (20). As there is currently no product available and no reimbursement for this indication in Germany, it will not be discussed further here. In view of the well-established, currently available and comparatively much cheaper PrEP options, the current price level of LA would certainly be a high barrier for demand in this special market segment.

### 4.5 Limitations

There are some limitations to this study and data source. Pharmacy prescription data are not person-specific and cannot be linked to other health insurance or treatment data, and therefore do not allow statistics on population characteristics or analysis of reasons for treatment decisions.

Our estimate based on the prescriptions can only be approximate for several reasons. Since the prescription data are only available monthly and the treatment regimens allow a deviation of 7 days around the target date, in quite a few cases, the prescription is likely to fall in a neighboring month. The prescriptions of 63 oral CAB and 23 CAB-LA suggested that an interval of 1 month was not maintained for 23 patients. Figure 1 also shows that the number of estimated prescriptions (columns) up to September 2022 lagged slightly behind the number of determined prescriptions (red line). It is conceivable that a small number of LA therapies were initiated without oral initiation. It is further recognizable that our idealized calculation without therapy discontinuation due to oral initiation does not completely coincide. After October 2022, the number of prescriptions is slightly lower than the estimated number of prescriptions in most months. Among other things, it is conceivable that some PLWHIV undergoing LA therapy have missed appointments for follow-up treatment, and oral CAB prescriptions have been used to bridge this gap. It is also imaginable that some PLWHIV dropped out of LA therapy again.

We conclude from the discrepancy between the 149 prescriptions of RPV-LA and CAB-LA that our data source itself is subject to a certain, albeit minor, degree of inaccuracy.

Nevertheless, due to the slight underestimation of the 14,523 prescriptions calculated by us compared to the 14,616 actual prescriptions, we are convinced that the oral initiation prescriptions currently form a good basis for determining the initiation of therapy.

## 5 Conclusion

To our knowledge, this is the first study of real-world use and the number of people receiving LA therapy. A strength of our study and the approach using pharmacy prescription data is the nearly complete coverage of people with statutory health insurance in Germany. Although the number of PLWHIV receiving LA therapy increased steadily, they accounted for just under 2% of the estimated total number of people receiving HIV therapy in Germany in 2023, almost 2 years after the market launch. Compared to the forecasted demand, the need for this mode of therapy in Germany appears to be limited. No significant increase in prescriptions is expected; on the contrary, the trend is leveling off and is unlikely to change drastically in the near future.

Implementation challenges and remaining unanswered questions may be a reason why PLWHIV and practitioners, when in doubt, stick with the long-established options of oral ART.

Follow-up studies at regular intervals on the further course would be useful and are recommended, as well as investigations into possible reasons for the slow uptake to inform public health experts and broaden treatment options.

## Data availability statement

The data analyzed in this study is subject to the following licenses/restrictions: Secondary data were purchased from Insight Health™ and license for further distribution applies. Aggregated data available by reasonable request to the corresponding author. Requests to access these datasets should be directed to DS, schmidtd@rki.de.

## Author contributions

DS: Conceptualization, Data curation, Formal analysis, Methodology, Validation, Visualization, Writing – original draft, Writing – review & editing. CK: Conceptualization, Data curation, Formal analysis, Methodology, Validation, Visualization, Writing – review & editing. MS: Conceptualization, Methodology, Validation, Writing – review & editing.
